# Photorespiratory glycolate oxidase is essential for the survival of the red alga *Cyanidioschyzon merolae* under ambient CO_2_ conditions

**DOI:** 10.1093/jxb/erw118

**Published:** 2016-03-19

**Authors:** Nadine Rademacher, Ramona Kern, Takayuki Fujiwara, Tabea Mettler-Altmann, Shin-ya Miyagishima, Martin Hagemann, Marion Eisenhut, Andreas P.M. Weber

**Affiliations:** ^1^Institute of Plant Biochemistry, Cluster of Excellence on Plant Sciences (CEPLAS), Heinrich Heine University, Universitätsstraße 1, 40225 Düsseldorf, Germany; ^2^University Rostock, Department Plant Physiology, Albert-Einstein-Straße 3, 18059 Rostock, Germany; ^3^Division of Symbiosis and Cell Evolution, National Institute of Genetics, 1111 Yata, Mishima 411–8540, Shizuoka, Japan; ^4^Japan Science and Technology Agency, CREST, 4-1-8 Honcho, Kawaguchi 332-0012, Saitama, Japan

**Keywords:** Evolution, glycolate oxidase, knockout mutant, metabolites, photorespiration, red alga.

## Abstract

Photorespiration is essential for all organisms performing oxygenic photosynthesis. The evolution of photorespiratory metabolism began among cyanobacteria and led to a highly compartmented pathway in plants. A molecular understanding of photorespiration in eukaryotic algae, such as glaucophytes, rhodophytes, and chlorophytes, is essential to unravel the evolution of this pathway. However, mechanistic detail of the photorespiratory pathway in red algae is scarce. The unicellular red alga *Cyanidioschyzon merolae* represents a model for the red lineage. Its genome is fully sequenced, and tools for targeted gene engineering are available. To study the function and importance of photorespiration in red algae, we chose glycolate oxidase (GOX) as the target. GOX catalyses the conversion of glycolate into glyoxylate, while hydrogen peroxide is generated as a side-product. The function of the candidate GOX from *C. merolae* was verified by the fact that recombinant GOX preferred glycolate over L-lactate as a substrate. Yellow fluorescent protein-GOX fusion proteins showed that GOX is targeted to peroxisomes in *C. merolae*. The GOX knockout mutant lines showed a high-carbon-requiring phenotype with decreased growth and reduced photosynthetic activity compared to the wild type under ambient air conditions. Metabolite analyses revealed glycolate and glycine accumulation in the mutant cells after a shift from high CO_2_ conditions to ambient air. In summary, or results demonstrate that photorespiratory metabolism is essential for red algae. The use of a peroxisomal GOX points to a high photorespiratory flux as an ancestral feature of all photosynthetic eukaryotes.

## Highlight

Glycolate oxidase knockouts in *Cyanidioschyzon* reveal that red algae harbour a plant-like photorespiratory pathway. This suggests that a photorespiratory pathway employing peroxisomal glycolate oxidase is ancient and not recently evolved.

## Introduction

The enzyme Rubisco catalyses the first step in photosynthetic carbon fixation by adding one molecule of carbon dioxide (CO_2_) to the acceptor molecule ribulose 1,5–bisphosphate (RuBP). The resulting two molecules of 3-phosphogylcerate (3-PGA) are fed into the Calvin–Benson cycle for the production of sugar molecules. Beside the carboxylation reaction, Rubisco also catalyses the oxygenation of RuBP in the presence of oxygen (O_2_). In this case, one molecule of 3-PGA and one of 2-phosphoglycolate (2-PG) are generated (Ogren and Bowes, 1971). 2-PG is detrimental to cellular metabolism, inhibiting enzymatic reactions such as those of triose-phosphate isomerase (Husic *et al.*, 1987; Norman and Colman, 1991). Thus, 2-PG is rapidly converted to 3-PGA by the photorespiratory pathway, which is distributed between the chloroplast, peroxisome, mitochondrion, and cytosol in plants (Somerville, 2001; Bauwe *et al.*, 2010). This pathway has nine enzymatic steps that convert two molecules of 2-PG into one molecule of 3-PGA at the expense of ATP and NADPH.

In addition to its metabolic repair function, photorespiratory metabolism is suggested to protect against acceptor limitation (Heber and Krause, 1980; Kozaki and Takeba, 1996; Takahashi *et al.*, 2007). The essential role of photorespiration under ambient CO_2_ conditions is demonstrated by the lethality of mutants with a completely impaired photorespiratory metabolism. These mutants display a photorespiratory, high-carbon-requiring (HCR) phenotype. That is, the mutants become chlorotic and fade under ambient, low CO_2_ concentrations. High CO_2_ concentrations typically suppress this phenotype (Somerville, 2001). So far, mutants displaying an HCR phenotype have been identified in plants, including *Zea mays* (Zelitch *et al.*, 2009) and *Arabidopsis thaliana* (Voll *et al.*, 2006; Engel *et al.*, 2007; Schwarte and Bauwe, 2007; Timm *et al.*, 2011), the green alga *Chlamydomonas reinhardtii* (Suzuki *et al.*, 1999; Nakamura *et al.*, 2005), and the cyanobacterium *Synechocystis* sp. strain PCC 6803 (Eisenhut *et al.*, 2008).

Photorespiration had already evolved in cyanobacteria, the first organisms performing oxygenic photosynthesis, and exists in all primary endosymbiotic lineages of algae as well as in plants today (Eisenhut *et al.*, 2008; reviewed in Bauwe *et al.*, 2010; Kern *et al.*, 2013). Owing to increasing O_2_ concentrations in the atmosphere, the photorespiratory pathway needed to be optimized to deal with the enhanced oxygenation activity of Rubisco and resulting enhanced flux through the pathway. This was achieved in the plant-type photorespiratory pathway by recruiting a glycolate oxidase (GOX) instead of a glycolate dehydrogenase (GlcD) for the photorespiratory glycolate-to-glyoxylate conversion (Kehlenbeck *et al.*, 1995). GOX has a much higher maximal rate (V_max_) than GlcD and is most efficient in an environment with a high O_2_ partial pressure, which enables the quick degradation of the GOX by-product hydrogen peroxide (H_2_O_2_) by catalase in the peroxisome (reviewed in Hagemann *et al.*, 2013). Thus, the use of GOX in peroxisomes can be considered an indicator of high photorespiratory flux in the optimized plant-type photorespiratory pathway (Kehlenbeck *et al.*, 1995; Hagemann *et al.*, 2013).

It has been postulated that photorespiratory metabolism is essential for all organisms that perform oxygenic photosynthesis and that it evolved very early among cyanobacteria (Eisenhut *et al.*, 2008; Bauwe *et al.*, 2010; Hagemann *et al.*, 2010; Hagemann *et al.*, 2016). However, to date no detailed studies on photorespiration in the other two branches of the Archaeplastida besides Chloroplastida, namely the Glaucophyta and Rhodophyta, have been performed. The red alga *Cyanidioschyzon merolae* serves as a model organism for the Rhodophyta. This unicellular alga is characterized by a simple eukaryotic cell structure with each cell having a single nucleus, mitochondrion, chloroplast, and peroxisome. Its small and minimally redundant 16 Mbp genome is completely known (Matsuzaki *et al.*, 2004) and methods for targeted gene knockout and protein localization are available (Ohnuma *et al.*, 2008; Imamura *et al.*, 2010)*. C. merolae* is an extremophile and can tolerate temperatures up to 57°C and pH values <2 (Seckbach, 1995). At this acidic pH, the vast majority of inorganic carbon is present as CO_2_ in the aquatic environment. Given that the acidophilic and acidotolerant algae manage to maintain a neutral pH inside the cells (Zenvirth *et al.*, 1985; Gimmler *et al.*, 1988; Colman and Balkos, 2005), the natural pH gradient must allow the diffusive uptake of CO_2_ into the cytoplasm, where CO_2_ is captured/accumulated in the form of HCO_3_^−^. A carbonic anhydrase recovers the CO_2_ and provides it to Rubisco for fixation. Importantly, red algal Rubisco has the highest specificity for CO_2_ over O_2_ (Uemura *et al.*, 1997) measured so far.

In this study we tested the hypothesis that photorespiratory metabolism is essential in the red alga *C. merolae.* We focused on red algal GOX, which also allowed us to investigate the hypothesis that plant-type photorespiratory metabolism evolved early in photosynthetic eukaryotes. To this end, we generated and physiologically characterized a mutant with the candidate GOX knocked out. The mutant displayed an HCR phenotype and accumulated glycolate upon a shift from high to low (ambient) CO_2_ conditions. Together with the findings that the enzyme localized to the peroxisomal matrix and that recombinant GOX displayed plant-like enzymatic features, we conclude that in the evolutionary basal lineage of red algae, the photorespiratory pathway is already functioning in the plant-type mode. This indicates a high photorespiratory flux before the colonization of terrestrial environments by photosynthetic eukaryotes.

## Material and methods

### Strains and culture conditions

*C. merolae* 10D was used as the wild-type (WT) strain in this study. For the generation of the Δ*gox* mutant, the M4 mutant, deficient in uracil synthesis, was used (Minoda *et al.*, 2004). Cells were cultivated in 2× modified Allen’s (2×MA) medium (pH 2; Minoda *et al.*, 2004) in glass vessels at 40°C, and aerated with high CO_2_ concentrations (5% CO_2_ in air, HC) or low CO_2_ concentrations (0.04% CO_2_ in air, LC) under continuous white light (90 µmol photons m^−2^ s^−1^). The growth medium for the M4 mutant was supplemented with 500 µg ml^−1^ uracil.

For the CO_2_ shift experiment, *C. merolae* WT and the Δ*gox* mutant strains were cultivated in a multicultivator system (Photon System Instruments, Drasov, Czech Republic) at 90 µmol photons m^−2^ s^−1^ light and 40°C. For growth rate calculation, OD_720_ measurements were performed every hour over the experimental time by the multicultivator system.

### Quantitative real-time PCR

Samples for RNA extraction were taken 0h, 3h, and 24h after the shift from HC to LC conditions, as well as 24h after the shift back to HC. RNA extraction was performed using the EURx GeneMatrix Universal RNA Purification Kit (Roboklon, Berlin, Germany) following manufacturer’s instruction for RNA cell extraction. DNase treatment was carried out using RQ1 RNA-Free DNase and cDNA synthesis was performed using M-MLV Reverse Transcriptase, RNase (H−), Point Mutant (Promega, Fitchburg, WI, USA). A MESA BLUE MasterMix for SYBR® Assay (Eurogentec, Seraing, Liège, Belgium) was employed for the quantitative (q)RT assay. The primers used for qRT-PCR were qRT-CmGOX-fw and qRT-CmGOX-rev (efficiency 2.0) for amplification of the *CmGOX* (*CMQ436C*) transcript and qRT-CmrbcL-fw and qRT-CmrbcL-rev (efficiency 2.1). The red algal homologue of the constitutively expressed gene *TIP-41-like* (*At4g34270*) in *A. thaliana* (Czechowski *et al.*, 2005) was used as a reference gene. We designated this gene, *CMM193C*, as *CmBLACK* and applied it as a reference for ΔΔCt analysis using the primers qRT-CmBlack-fw and qRT-CmBlack-rev (efficiency 2.2). Primer sequences are listed in Supplementary Table S1. qRT-PCR was performed with the StepOne Plus Real-Time system (Applied Biosystems, Waltham, MA, USA). Mean normalized expression was calculated from three biological replicates, including three technical replicates, following the instructions of Simon (2003).

### Subcellular localization studies

The CmGOX coding sequence was amplified using Phusion High Fidelity DNA Polymerase (New England Biolabs, Ipswich, MA, USA), P1 forward primer (*Mfe*I restriction site), P2 reverse primer (*Nco*I restriction site), and *C. merolae* genomic DNA as the template. The PCR product was ligated into the pJET2.1 vector system (ThermoFisher Scientific, Waltham, MA, USA) for amplification and sequencing. For localization studies in *Nicotiana benthamiana*, pUBN-YFP-Dest (Grefen *et al.*, 2010) was used as the final vector for fusion of CmGOX with an N-terminal yellow fluorescent protein (YFP) tag. Expression of the fusion protein was under the control of the constitutive *UBIQUITIN 10* promoter. Transformation of *N. benthamiana* was carried out using *Agrobacterium tumefaciens* strain GV3101. For peroxisomal co-localization studies, *A. tumefaciens* cells containing a vector for expression of the cyan fluorescent protein (CFP) fused with the C-terminal peroxisomal target signal 1 (PTS1) were co-infiltrated into the leaf. The CFP::PTS1 construct was used as a peroxisomal-targeted fluorescent marker (Linka *et al.*, 2008). Protoplast isolation and microscopic analysis were performed 2 d after infiltration using a Zeiss LSM 510 Meta laser microscope as described in Breuers *et al.* (2012).

For localization studies in *C. merolae*, the *UBIQUITIN 10* promoter of the pUBN-YFP-Dest vector was exchanged for the *apcC* promoter. To this end, the *apcC* promoter region was synthesized by Phusion PCR using the pCG1 vector (Watanabe *et al.*, 2011) as the template and the primer P3 (*Pme*I restriction site) and P4 (*Spe*I restriction site). The vector pJET2.1 (ThermoFisher Scientific) was used for subcloning. Transformation of *C. merolae* was performed as described by Ohnuma *et al.* (2008). Microscopic analysis was carried out 1 d after transformation using the Zeiss LSM 780 laser microscope. Primer sequences are listed in Supplementary Table S1.

### Δgox mutant generation

To inactivate the *CmGOX* gene, the *CmGOX* (*CMQ436C*) locus was replaced by the *C. merolae URA* gene by homologous recombination. The *CmGOX* genomic region was amplified with the primers GOX_KO_F1 and GOX_KO_R1. The amplified DNA was cloned into the pQE80 vector (Qiagen, Hilden, Germany) using the In-Fusion HD Cloning Kit (Clontech, Mountain View, CA, USA). The 3′ portion, vector, and 5′ portion of *CmGOX* was amplified with the primers GOX_KO_F2 and GOX_KO_R2 and then the *URA* gene amplified with URA_F and URA_R was inserted by In-Fusion Cloning. The *CmGOX* genomic region with the *URA* insert was amplified from the vector by PCR with primers GOX_KO_F3 and GOX_KO_R3 and was transformed into *C. merolae* M4, a derivative of *C. merolae* 10D, which has a mutation in the *URA* gene (Minoda *et al.*, 2004). Transformation and selection of the gene knockouts were performed as described (Imamura *et al.*, 2010; Fujiwara *et al.*, 2015). The occurrence of the recombination events in the *CmGOX*-knockout strains was confirmed by PCR with the primer sets GOX_KO_F4/GOX_KO_R4 and GOX_KO_F5/GOX_KO_R5. Absence of *CmGOX* transcripts in the knockout lines was verified by RT-PCR analysis using the primers GOX_KO_F2 and GOX_KO_R2. Transcripts from the *CMQ432C* locus adjacent to *CmGOX* were amplified using the primers CMQ432C-F and CMQ432C-R as a control. Primer sequences are given in Supplementary Table S1.

### Metabolite extraction and analysis

Centrifugation was used to harvest 10ml cells (4°C, 5min, 3000 RCF) and the resulting pellets were frozen in liquid nitrogen. For metabolite extraction, the pellets were resuspended in ethanol (70% v/v, including 50 µM ribitol as internal standard), using acid-washed glass beads for cell disruption by vortexing (3×1min, 4°C, maximum speed). The extraction mix was centrifuged (4°C, 2min, 16000 RCF) and the supernatant was analysed by gas chromatography coupled to a time-of-flight mass spectrometer (7200 GC-QTOF; Agilent Technologies, Santa Clara, CA, USA) according to Fiehn and Kind (2007). Peak areas for all compounds were analysed using the Mass Hunter Software (Agilent) and curated manually if necessary.

For the analysis of glycine and serine, the same extract was derivatized by AccQ-Tag Ultra Reagent Powder (Waters Corporation, Milford, MA, USA) according to the manufacturer’s instructions and separated at 60°C and a flow of 0.7ml min^−1^ on an AccQ-Tag™ Ultra Column 2.1×100mm and particle size 1.7 μm (Waters Corporation) using the 10% AccQ-Tag™ Ultra Eluent A (Waters Corporation) and acetonitrile as eluent B (0–0.54min, 0.1% B; 0.54–5.74min, 0.1–9.1% B; 5.74–7.74min, 9.1–21.2% B; 7.74–8.04min, 21.2–59.6% B and maintained for 0.6min; 8.64–8.73min, 59.6–95% B and maintained for 0.27min; 9.0–9.1min, 95–100% B and maintained for 0.1min). Derivatized compounds were detected at 260nm. For this purpose a 1290 UHPLC system coupled to diode array detector from Agilent was used.

### Photosynthetic rate measurement

Cultivation of WT and the Δ*gox* #46 mutant was performed under continuous light (80 µmol photons m^−2^ s^−1^) at 28°C. Cells were pre-cultivated at 5% CO_2_. Twenty-four hours before measurements, cultures were adjusted to a cell density of OD_750_ of 0.7 and shifted to either HC (bubbling with 5% CO_2_) or LC conditions (bubbling with ambient air containing 0.04% CO_2_). Cells were then collected by centrifugation (4000rpm, 5min, room temperature), washed, and resuspended in CO_2_-free 2×MA growth medium to a final OD_750_ of 1. The oxygen production of 3ml of this cell culture was used to quantify the photosynthetic rate at 28°C and a saturating light intensity of 120 µmol photons m^−2^ s^−1^, using a Clark electrode (Oxygraph System; Hansatech, Norfolk, England). To determine the CO_2_-dependent photosynthetic rate, sodium hydrogen bicarbonate (NaHCO_3_) was stepwise added to a final saturating concentration of 257 µM. Oxygraph Plus software (Hansatech) and Prism (GraphPad Software, La Jolla, CA, USA) was used for data analysis.

### Chlorophyll *a* determination

To analyse chlorophyll *a* (Chl *a*) concentration, 1ml of cell culture was centrifuged (2min, 16 000 RCF, room temperature) and 900 µl supernatant was exchanged by methanol (100% v/v). The suspension was incubated for 10min at 65°C and measured at OD_665_. The extinction value (extinction coefficient of 78.74l g^−1^ cm^−1^) was multiplied by 12.7 to calculate the Chl *a* content in micrograms per millilitre (Meeks and Castenholz, 1971).

### Heterologous expression of CmGOX and enzyme assay

To generate a CmGOX overexpressing *Escherichia coli* strain, the coding sequence was amplified by PCR using genomic DNA from *C. merolae*, gene-specific primers with added cleavage sites (CMQ436C-EcoRI-fw and CMQ436C-SalI-rv; Supplementary Table S1), Taq Polymerase Mastermix (Qiagen), and proof-reading Elongase enzyme (ThermoFisher Scientific). The resulting 1182bp fragment was cloned into the pGemT-vector (Promega). After sequence confirmation (Seqlab, Göttingen, Germany), the coding sequence was cloned into the expression vector IBA43+ in frame with the N-terminal His-tag using *Eco*RI/*Sal*I and subsequently transformed into the *E. coli* strain BL21 DE3. To improve the folding of the recombinant protein, the CmGOX-overexpressing *E. coli* strain was co-transformed with the pG-KJE8 plasmid coding for five chaperones (Takara, Ohtsu, Japan).

*E. coli* BL21 cells containing IBA43+-CmGOX and pG-KJE8 were grown in lysogeny broth medium supplemented with plasmid-specific antibiotics, l-arabinose, and tetracycline (0.05% and 0.5ng ml^−1^ final concentration) to an OD_750_ of 0.6 at 30°C. CmGOX expression was induced by the addition of 200 µg l^−1^ anhydrotetracycline and the cells were incubated for 16h at 30°C. The fusion protein was purified via the N-terminal His-tag using Ni-NTA Sepharose according to the protocol of the supplier (ThermoFisher Scientific). All purification steps were performed at 4°C. The cells were harvested and resuspended in homogenization buffer (20mM Tris-HCl, pH 8.0, containing 500mM NaCl, 1mM DTT, and 0.1mM FMN). Proteins were extracted by ultrasonic treatments (4×30s, 90W) in ice. Soluble protein extracts were used for affinity chromatography on Ni-NTA Sepharose using the homogenization buffer supplemented with 40–80mM imidazole as washing buffers. The elution buffer contained 300mM imidazole. The elution fractions were combined and desalted using PD-10 columns (GE Healthcare, Little Chalfont, UK). Finally, the recombinant enzymes were dissolved in 20mM Tris-HCl, pH 8.0, containing 1mM DTT, and 0.1mM FMN. The eluted proteins were checked regarding purity using SDS-PAGE. Protein concentration was determined using Bradford’s method with bovine albumin as the standard protein (Bradford, 1976).

The purified and desalted recombinant His-tagged protein was used for enzyme assays with a Hansatech oxygen electrode as described in detail by Hackenberg *et al.* (2011).

## Results

### The genome of *C. merolae* contains single copy genes for all enzymes of the plant-type photorespiratory pathway

**Fig. 1.**
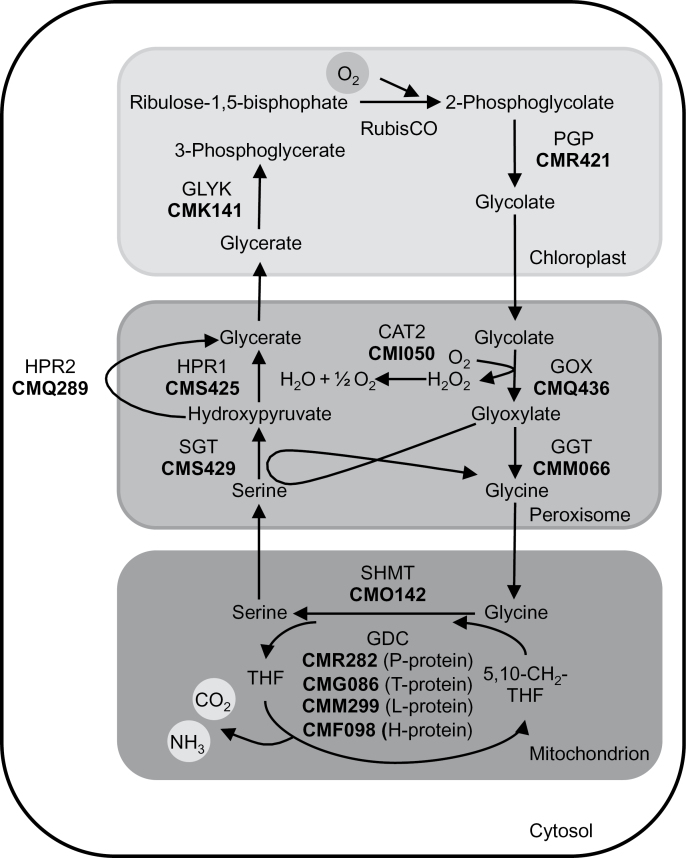
Schematic view of the photorespiratory pathway in *C. merolae*. Candidate proteins in *C. merolae* were identified by BLAST analysis. Corresponding protein identifiers (acc. to *C. merolae* Genome Project, http://merolae.biol.s.u-tokyo.ac.jp/; Matsuzaki *et al.*, 2004) are shown in bold. The enzymes are CAT2: catalase 2; GDC: glycine decarboxylase; GGT: glutamate:glyoxylate aminotransferase; GLYK, glycerate 3-kinase; GOX, glycolate oxidase; HPR1, peroxisomal hydroxypyruvate reductase 1; HPR2, cytosolic hydroxypyruvate reductase 2; PGP, 2-PG phosphatase; Rubisco, ribulose-1,5-bisphosphate carboxylase/oxygenase; SHMT, serine hydroxymethyl transferase; SGT, serine:glyoxylate aminotransferase.

We first performed BLASTP analyses using the BLAST tools of both the *C. merolae* Genome Project (http://merolae.biol.s.u-tokyo.ac.jp/blast/blast.html; Matsuzaki *et al.*, 2004) and the National Center for Biotechnology Information (http://blast.ncbi.nlm.nih.gov/Blast.cgi), to identify in *C. merolae* proteins homologous to the photorespiratory enzymes in *A. thaliana*. The query and match proteins from *A. thaliana* and *C. merolae*, respectively, are listed in Supplementary Table S2. As a result, we identified the full protein repertoire of a plant-type photorespiratory pathway in *C. merolae* (Fig. 1). In contrast to *A. thaliana* and other land plants, single copy genes and not gene families encode the photorespiratory enzymes. For the majority, the *A. thaliana* query and the identified *C. merolae* proteins were the best reciprocal BLAST hits. We were unable to identify a protein homologous with the glycolate dehydrogenase that acts in the photorespiratory pathway of *Chlorophyta* such as *C. reinhardtii* (Nakamura *et al.*, 2005). This suggests that the glycolate-to-glyoxylate converting step is probably catalysed by GOX in *Rhodophyta* such as *C. merolae*. In accordance with the predicted GOX activity, we identified a catalase (Fig. 1) that is needed to decompose the H_2_O_2_, which is generated as a side-product of GOX activity.

### CmGOX has a higher affinity for glycolate than l-lactate

**Fig. 2.**
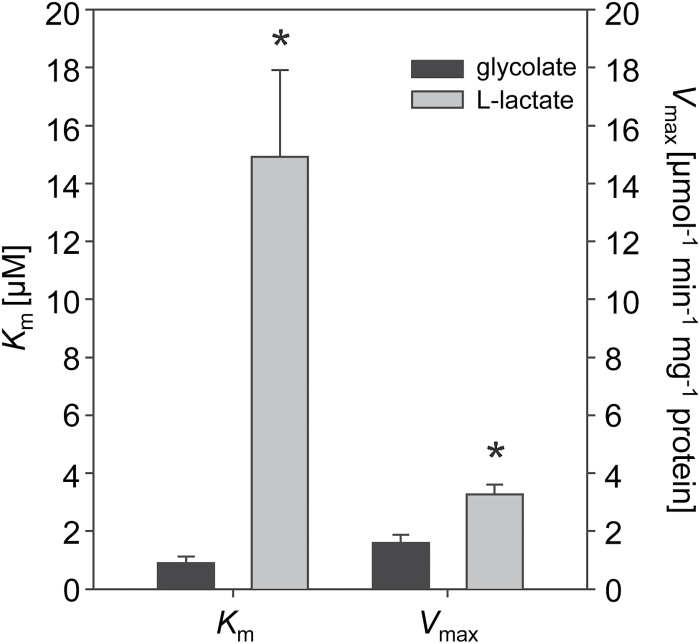
Biochemical characterization of recombinant CmGOX. The *K*_m_ and *V*_max_ values of CmGOX for the substrates glycolate and l-lactate were calculated by non-linear regression following the Michaelis–Menten kinetic implemented in SigmaPlot 11.0. The means of at least two independent enzyme preparations ± SD are given. Asterisks indicate significant differences (*P* < 0.05) determined with the two-tailed Student’s t-test.

To verify the enzymatic activity of the putative GOX from *C. merolae,* the coding gene was heterologously expressed in *E. coli*. The purified His-tagged protein (see Supplementary Fig. S1) was used for enzymatic assays, in which the O_2_ consumption was determined depending on the substrates l-lactate or glycolate. Like the homologous protein from the plant *A. thaliana*, CmGOX catalysed the oxidation of both substrates, l-lactate and glycolate (Fig. 2). The low *K*_m_ value for glycolate (0.9±0.2mM) and significantly higher *K*_m_ value for l-lactate (14.9±3.0 µM) clearly support the hypothesis that the gene *CMQ436C* encodes an oxidase with higher affinity for glycolate than l-lactate. The *V*_max_ for l-lactate (3.3±0.3 µmol min^−1^ mg^−1^) was twice as high as that for glycolate (1.5±0.3 µmol min^−1^ mg^−1^). The occurrence of GOX in *C. merolae* suggests high flux through the photorespiratory pathway, as found in vascular plants.

### CmGOX is localized in the matrix of peroxisomes

**Fig. 3.**
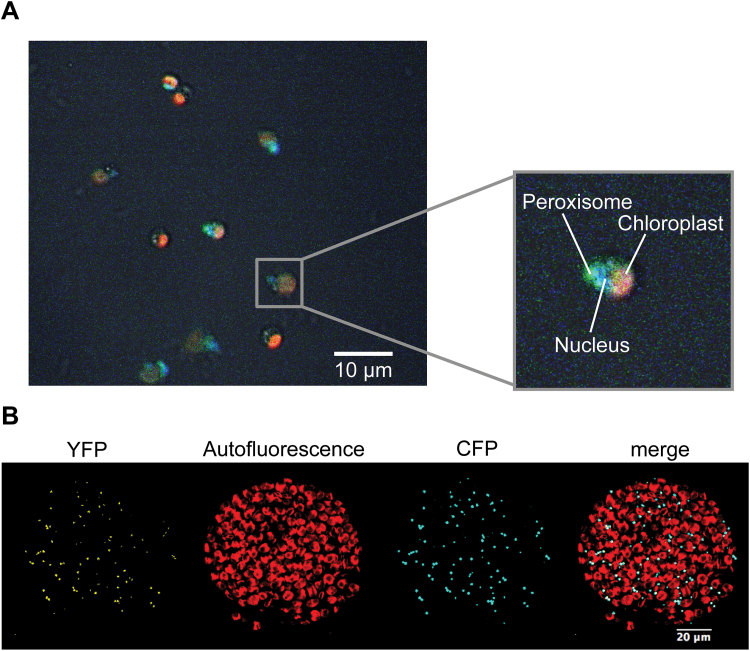
Subcellular localization studies of CmGOX. (**A**) Localization of CmGOX in *C. merolae.* Red: chloroplast autofluorescence; blue: DAPI-stained nucleus; green: YFP signal from YFP::CmGOX construct*. C. merolae* cells were transformed 24h before microscopic analysis. (**B**) Localization of CmGOX in tobacco protoplasts. From left to right: YFP signal of YFP::CmGOX construct, chlorophyll autofluorescence, CFP signal as peroxisomal marker (CFP::PTS1), and merge of all three pictures. Microscopic analyses were performed with protoplasts isolated from transiently transformed *N. benthamiana* leaves.

Typically, GOX proteins reside in the peroxisomal matrix, where the critical GOX catalysis by-product H_2_O_2_ is efficiently decomposed by the activity of catalase. The CmGOX protein sequence contains a putative peroxisome targeting sequence (SKL) at the C-terminus (Matsuzaki *et al.*, 2004). To experimentally determine the actual subcellular localization, we generated GOX protein variants that were fused with an N-terminal YFP-domain. For expression in *C. merolae* cells, the YFP::CmGOX construct was set under the control of the strong *apcC* promoter, which has been shown to be suitable for protein localization studies in *C. merolae* (Watanabe *et al.*, 2011). Fluorescence microscopy demonstrated that the YFP::CmGOX resides in the peroxisome in *C. merolae* (Fig. 3A). To improve the resolution, we alternatively expressed the YFP::CmGOX construct under control of the *UBIQUITIN 10* promoter in tobacco leaves. The overlap of the peroxisomal marker (CFP::PTS1, Linka *et al.*, 2008) signal and the YFP::CmGOX signal in protoplasts confirmed the localization of CmGOX in the peroxisomal matrix (Fig. 3B).

### The amount of *CmGOX* transcript increases under LC conditions

**Fig. 4.**
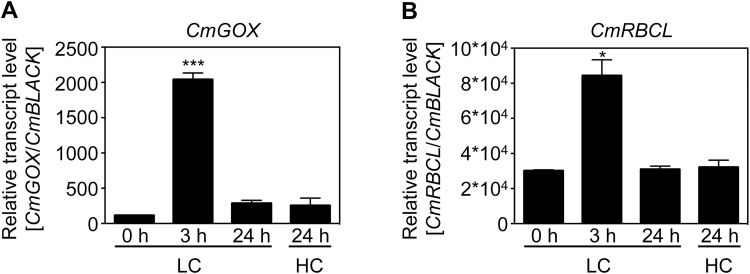
Effect of shift in CO_2_ concentrations on transcript levels of *CmGOX* (**A**) and *CmRBCL* (**B**). Samples were taken before a shift from HC (5% CO_2_) to LC (0.04% CO_2_) conditions, 3h and 24h after the shift, and 24h after a shift back to HC conditions. Shown is the mean normalized expression of three biological replicates including three technical replicates and the mean normalized standard error. *CmBLACK* (*CMM193C*) was used as the reference gene for Ct analysis. Significant differences to initial transcript levels (LC, 0h) were analysed by a two-tailed Student’s t-test (***P < 0.001).

To examine if transcript amounts of *CmGOX* responded to changes in CO_2_ availability, the *CmGOX* steady state transcript level was analysed in *C. merolae* WT cells cultivated under photorespiration-suppressing HC conditions and after a shift to photorespiration-stimulating LC conditions. *CmGOX* transcript abundance increased 100-fold 3h after the shift to LC in comparison to constant HC conditions. After 24h under LC conditions, the *CmGOX* transcript level returned to the low amounts typical of HC-grown cells (Fig. 4A). We observed a comparable abundance pattern for the gene encoding the large subunit of Rubisco (*CmRBCL*). The transcript level significantly increased 3-fold 3h after the shift to LC, while it was similar to the HC value after 24h in LC conditions (Fig. 4B).

### Deletion of *CmGOX* causes an HCR phenotype and perturbations in photorespiratory metabolism

**Fig. 5.**
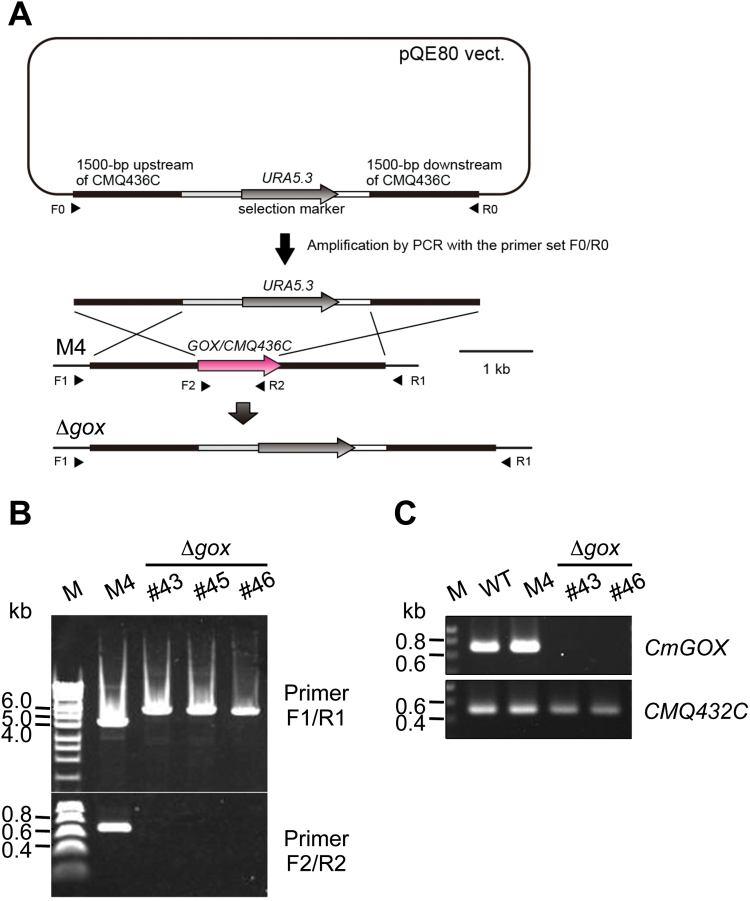
Generation of a Δ*gox* knockout mutant in *C. merolae*. (**A**) Schematic presentation of the strategy to generate a knockout mutant for *CmGOX*. For detailed information see ‘Materials and methods’. (**B**) Verification of Δ*gox* mutant lines #43, #45, and #46. PCR was performed on genomic DNA of the M4 background mutant and the Δ*gox* mutant lines #43, #45, and #46 with primers flanking the *CmGOX* upstream and downstream regions (F1/R1) and the *CmGOX* coding region (F2/R2), respectively. Expected fragment sizes were F1/R1: 4.5kb (M4), 6kb (Δ*gox*); F2/R2: 0.66kb (M4), – (Δ*gox*). (**C**) Verification of absence of *CmGOX* transcripts in the Δ*gox* knockout lines #43 and #46. RT-PCR analysis was performed on cDNA isolated from WT, M4 background mutant, and the Δ*gox* mutant lines #43 and #46 with primers flanking the *CmGOX* coding region (F2/R2). Transcripts from the *CMQ432C* locus adjacent to the *CmGOX* locus were used as a control. Expected fragment sizes are *CmGOX*: 698bp; *CMQ432C*: 520bp.

To study the importance of photorespiration and especially the function of the GOX protein for *C. merolae,* we generated Δ*gox* knockout mutant lines. Mutant generation was performed as described by Imamura *et al.* (2010). The *CmGOX* coding sequence was exchanged for the *URA5.3* marker gene via homologous recombination (Fig. 5A), conferring uracil autotrophy to the otherwise uracil auxotrophic M4 mutant strain. Correct recombination events were verified for the mutant lines Δ*gox* #43, #45, and #46 by PCR using genomic DNA as the template (Fig. 5B). Because *CmGOX* transcripts could not be detected for Δ*gox* #43 and Δ*gox* #46 (Fig. 5C) at the mRNA level, we chose these as the knockout mutant lines for further analyses.

**Fig. 6.**
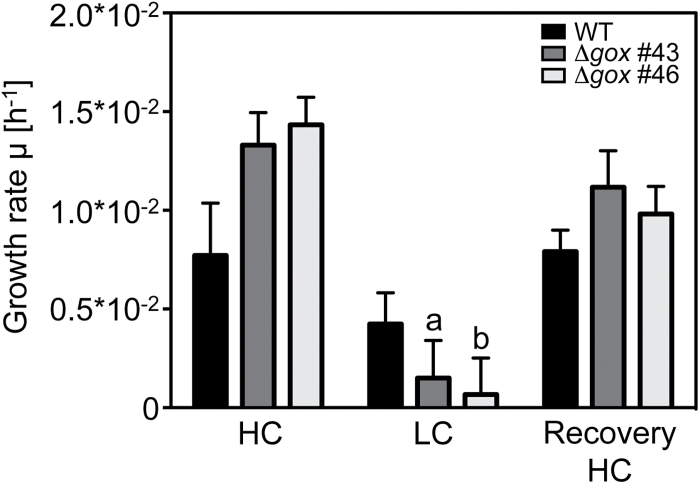
Growth rate of WT cells and Δ*gox* mutant line #43 and #46 grown for 24h under HC conditions (5% CO_2_), 24h under LC conditions (0.04% CO_2_), and returned for 24h to HC conditions. OD_720_ measurements were performed every hour by the Multi-Cultivator system (Photons System Instruments). Shown are means of three biological replicates with standard error. Significant differences to initial growth (HC) within one cell line were determined with a two-tailed Student’s t-test and are indicated by *a* (*P* < 0.01) and *b* (*P* < 0.001). Significant differences between the mutant and corresponding WT values could not be detected by t-test.

To gain insight into the impact of *CmGOX* deletion on the metabolism of *C. merolae* we performed a CO_2_ shift experiment, in which WT and the knockout mutant lines Δ*gox* #43 and Δ*gox* #46 were pre-cultivated under HC conditions, shifted to LC conditions for 24h, and then shifted back to HC conditions for another 24h. Under HC conditions, the growth performance of WT and mutants was not significantly different (Fig. 6). The shift towards LC conditions led to almost fully impaired growth in the Δ*gox* #43 and Δ*gox* #46 mutants, whereas WT cells continued growing. After the shift back to HC conditions, all cultures resumed growth and no difference in growth rates could be detected (Fig. 6). Chl *a* concentrations did not significantly differ between WT and mutant cells (Supplementary Fig. S2). Reduced growth of the Δ*gox* #43 and Δ*gox* #46 mutants was also observed when they were grown on solid medium under LC compared to HC conditions (Supplementary Fig. S3). Thus, the deletion of *CmGOX* resulted in an HCR phenotype, indicating an important role for GOX activity under ambient air conditions in *C. merolae*.

**Fig. 7.**
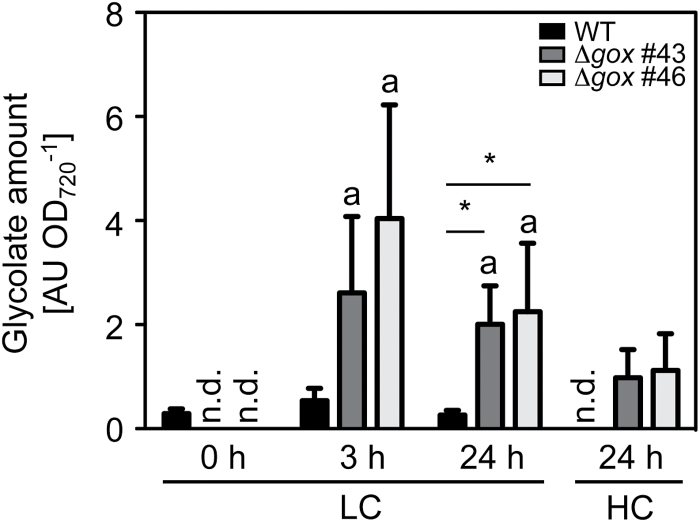
Glycolate levels of WT and Δ*gox* mutants #43 and #46 during the CO_2_ shift experiment. Glycolate levels of the different strains were determined by GC-MS before the shift from HC (5% CO_2_) to LC (0.04% CO_2_) conditions, 3h and 24h after the shift to LC, and after a 24h recovery phase under HC conditions. Shown are means of four biological replicates and standard errors. n.d., not detectable. Significant differences were analysed by the non-parametric Mann–Whitney test. Significant differences to initial glycolate levels (HC) within one cell line are indicated by *a* (*P* < 0.05). Significant differences between the mutant and corresponding WT values are indicated by an asterisk (*P* < 0.05).

During the shift experiment we took samples and performed a targeted metabolite analysis. Samples were taken before (0h), 3h, and 24h after the shift to LC, and 24h after the re-shift to HC conditions. When grown continuously under HC conditions, glycolate levels were below the detection limit. Importantly, 3h after the shift to LC, glycolate had accumulated to a level at which it could be measured. Its concentration was two to four times higher in Δ*gox* mutant lines than in WT. After 24h at LC, glycolate levels declined slightly in all cultures but were still significantly elevated in the mutants compared to WT. When the cultures were allowed to recover for 24h under HC conditions, glycolate was only detectable in the Δ*gox* mutant lines but not in WT cells (Fig. 7). Glycine-to-serine conversion in mitochondria is a central step in the photorespiratory pathway (see Fig. 1). In the WT we observed significant increases in both the glycine and the serine levels 3h after the shift to LC conditions (Supplementary Fig. S4A, B). The Δ*gox* mutants showed a different metabolic response. Glycine concentration was already higher under HC conditions in the mutants cells compared to WT, and increased by 2-fold 3h after the shift to LC, and by 3-fold 24h after the shift. After 24h recovery under HC conditions, glycine levels were still elevated in the mutants (Supplementary Fig. S4A). Serine levels had the opposite response and were lower in the Δ*gox* #43 and Δ*gox* #46 mutants than in the WT. A statistically significant difference was observed 3h after the LC shift, with serine levels of only 40% compared to the WT levels (Supplementary Fig. S4B). For the photorespiratory intermediate glycerate we did not detect significant differences between WT and mutant lines (Supplementary Fig. S4C). With respect to sugars we detected a significant decline in glucose levels 24h after LC treatment in all strains. After 24h HC treatment, the values fully recovered (Supplementary Fig. S4D). Fructose concentrations were also reduced in the WT 24h after the shift to LC conditions, but were not affected in the mutant lines by changes in CO_2_ availability (Supplementary Fig. S4E).

### The Δ*gox* mutant has inhibited photosynthetic activity

**Fig. 8.**
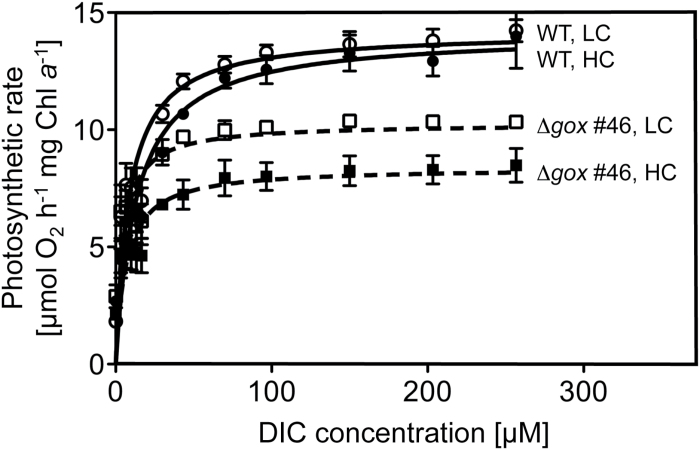
Photosynthesis rates of WT and Δ*gox* #46 mutant at increasing concentrations of externally supplied dissolved inorganic carbon (DIC). Cells were cultivated under HC conditions (5% CO_2_) and O_2_ evolution was measured using a Clark electrode. For LC measurements, cells were shifted for 24h to LC conditions (0.04% CO_2_). The photosynthetic rate was analysed according to increasing DIC concentration in the medium. Fitting analysis was performed using the Michaelis–Menten Kinetic (Prism 5) on the basis of three biological replicates each.

We investigated the impact of *CmGOX* deletion on photosynthetic activity by determining O_2_ production in increasing HCO_3_^−^ concentrations (Fig. 8). WT cells grown under either HC or LC conditions had a *V*_max_ of 14 µmol O_2_ h^−1^ per mg Chl *a* (Table 1). However, WT cells grown under HC conditions had a higher apparent *K*_m_ (*K*_m_ = 13.9 µM) compared to WT cells grown under LC conditions (*K*_m_ = 9.1 µM), which means that LC-grown cells were quicker to reach the maximal photosynthetic rate (Table 1). However, this difference was not significant. In comparison to WT, the Δ*gox* #46 mutant line showed a significantly lower maximal photosynthetic rate, and reduced but not significantly different *K*_m_ values (Table 1, Fig. 8). Mutant cells grown under LC conditions had a higher *V*_max_ (*V*_max_ = 10.3 µmol O_2_ h^−1^ per mg Chl *a*) than mutant cells grown under HC conditions (*V*_max_ = 8.6 µmol O_2_ h^−1^ per mg Chl *a*). The different growth conditions resulted in different *K*_m_ values (HC *K*_m_ = 8.4 µM; LC *K*_m_ = 4.9 µM; Table 1), as was the case for the WT.

**Table 1.** Effect of CO_2_ concentration on photosynthetic rates (*V*_max_) and C_i_ affinities (*K*_m_) of WT and Δ*gox* #46 cells.

	HC		LC	
	WT	Δ*gox* #46	WT	Δ*gox* #46
*V*_max_ [µmol O_2_ h^−1^ Mg^−1^ Chl *a*]	14.12±0.49	**8.58±1.62**	14.20±0.49	**10.31±0.60**
*K*_m_ [µM]	13.94±4.75	8.42±7.69	9.069±3.60	4.85±2.68

Results are presented as mean values ± SD from three independent determinations each. Significant differences (two-tailed Student’s t-test, *P* < 0.01) between WT and Δ*gox* #46 mutant are given in bold. Within one line results for HC and LC conditions were not significantly different.

## Discussion

The red alga *C. merolae* lives in acidic and hot aquatic habitats, which are naturally low in CO_2_. The solubility of CO_2_ and its diffusion coefficient is up to four magnitudes lower in water than in air (reviewed in Moroney *et al.*, 2013). Consequently, the red algal Rubisco evolved a characteristically high specificity for CO_2_, which is indicative of low CO_2_ concentrations at the site of Rubisco activity (Savir *et al.*, 2010). Despite its optimized Rubisco, red algae have been suggested to execute a carbon concentrating mechanism (CCM) that enhances the CO_2_ concentration near Rubisco (Zenvirth *et al.*, 1985; Giordano *et al.*, 2005). This raises the question of whether the survival of *C. merolae* under ambient conditions depends on photorespiratory metabolism. The importance of photorespiration in this organism has not been investigated to date.

The red alga *C. merolae* harbours a photorespiratory pathway that appears to be more similar to that of land plants than to *C. reinhardtii,* as revealed by BLAST analysis. All photorespiratory enzymes known from land plants, including catalase, are encoded in the genome of *C. merolae* and are homologous to the plant-type photorespiratory proteins (Supplementary Table S2). In contrast to land plants, all enzymes are encoded by single genes in the small and minimally redundant genome of *C. merolae*. Besides the enzymes catalysing the conversion of the photorespiratory intermediates, transporters shuttling the intermediates between the different organelles play a central role in photorespiratory metabolism in photosynthetic eukaryotes (reviewed in Eisenhut *et al.*, 2013). Among the few photorespiratory transporters identified to date, only the plastidic glycolate glycerate translocator PLGG1 (Pick *et al.*, 2013) is encoded in the genome of *C. merolae.* Homologues for the two plastidic carboxylate translocators DiT1 and DiT2 (Weber and Flügge, 2002; Renné *et al.*, 2003), which are involved in photorespiratory nitrogen recycling, do not exist in *C. merolae* (reviewed in Eisenhut *et al.*, 2015). We did not identify any proteins in *C. merolae* that were homologous with enzymes of the bacterial-type glycerate pathway, as found in cyanobacteria (Eisenhut *et al.*, 2006). We were also unable to identify a GlcD-type glycolate dehydrogenase. Thus, we hypothesized that the candidate GOX protein identified in our study catalyses the conversion of glycolate to glyoxylate in *C. merolae*, which supports previous phylogenetic analyses by Kern *et al.* (2013).

Our analysis revealed that *C. merolae* and probably all *Rhodophyta* perform a plant-like glycolate-to-glyoxylate conversion via a specific GOX in the peroxisome. Heterologous expression of the candidate GOX from *C. merolae* revealed a higher affinity for glycolate than for l-lactate (Fig. 2), and thus supported the likely usage of a GOX for the conversion of glycolate to glyoxylate during photorespiration. We could furthermore demonstrate that, as in plants, CmGOX resides in the peroxisome (Fig. 3), which is a prerequisite for it to function as a photorespiratory GOX. Given that the employment of a GOX is indicative of high flux through the photorespiratory pathway (Kehlenbeck *et al.*, 1995; Hagemann *et al.*, 2013), we suggest that, similar to plants, photorespiratory flux is high in the red alga *C. merolae*. In has previously been assumed that algae such as *C. reinhardtii*, which use a CCM, are characterized by low photorespiratory flux rates (Birmingham *et al.*, 1982). Indeed, glycolate-to-glyoxylate conversion by GlcD in the mitochondria of *C. reinhardtii* meets these low-flux requirements (Kehlenbeck *et al.*, 1995; Nakamura *et al.*, 2005). Moreover, in *Chlorophyceae* such as *C. reinhardtii*, the reduction of hydroxypyruvate and the transaminase steps occur in the mitochondria, and not in the peroxisomes (Atteia *et al.*, 2009). Accordingly, peroxisomes do not seem to be involved in photorespiration in *Chlorophyceae*, which could be the result of a different peroxisomal enzyme repertoire in this algal lineage (Stabenau, 1974; Stabenau *et al.*, 1993).

Further support for the involvement of CmGOX in the photorespiratory pathway in *C. merolae* was provided by gene expression analysis. *CmGOX* transcript amounts strongly increased 3h after the shift to photorespiratory conditions (Fig. 4A). The 100-fold increase of the *CmGOX* transcript level coincided with accumulation of glycolate in WT cells 3h after the shift to LC conditions (Fig. 7). It must be mentioned that an increase in transcript abundance does not necessarily lead to a proportional increase in protein abundance, as, for example, demonstrated for *C. reinhardtii* (Mettler *et al.*, 2014). However, the transcriptional response indicates that *C. merolae* quickly senses a change in the CO_2_ environment. Comparably, genes for CCM components and photorespiratory enzymes are upregulated in the chlorophyte *C. reinhardtii* (Fang *et al.*, 2012). However, it is not known how oxygenic phototrophs sense alterations in CO_2_ concentrations (Raven, 2006). The accumulation of photorespiratory intermediates such as 2-PG (Haimovich-Dayan *et al.*, 2015) and glycolate (Hackenberg *et al.*, 2012) could serve as signals that trigger the enhanced expression of the CCM genes among cyanobacteria under LC conditions.

The plant-like photorespiratory GOX is essential for survival of *C. merolae* under ambient conditions, as demonstrated by the occurrence of the HCR phenotype in the Δ*gox* knockout mutants. Mutant growth almost completely stopped after a shift from 5% CO_2_ to ambient air, whereas it quickly recovered when mutant cells were shifted back to HC conditions (Fig. 6, Supplementary Fig. S3). This behaviour is typical for the HCR phenotype (Somerville, 2001). However, in contrast to a plant HCR phenotype, we did not observe a chlorotic phenotype in the Δ*gox* mutants after the shift to LC conditions (Supplementary Fig. S2). A further argument for the function of photorespiratory GOX is the accumulation of glycolate in the Δ*gox* knockout mutant lines under LC conditions. A 24h recovery phase in HC was not sufficient to reduce the amount of glycolate below detectable levels in the Δ*gox* mutants, in contrast to what was observed for the WT (Fig. 7). Similarly, the GlcD mutant in *C. reinhardtii* showed a 4-fold higher accumulation of glycolate compared to the WT (Nakamura *et al.*, 2005). The same was true for a GOX mutant in *Zea mays*, which showed an 11-fold higher glycolate level after 25h in ambient air compared to the stable glycolate level in WT plants (Zelitch *et al.*, 2009).

Furthermore, the photosynthetic performance of Δ*gox* knockout mutants was affected (Fig. 8, Table 1). WT cells showed similar maximal photosynthetic rates when grown under HC or LC conditions, but enhanced CO_2_ affinity was found under LC conditions, as has been previously reported (Zenvirth *et al.*, 1985). Although not significant, cells of the mutant Δ*gox* #46 showed an enhanced affinity towards CO_2_ compared to the WT cells. However, maximal photosynthetic activity was significantly reduced in the mutant, which is likely the result of toxic effects due to the impaired photorespiratory pathway. For example, accumulation of the photorespiratory intermediates 2-PG (Norman and Colman, 1991) and glycine (Eisenhut *et al.*, 2007) is known to inhibit growth and photosynthesis.

In conclusion, all obtained results support the hypothesis that *C. merolae* has a plant-type photorespiratory pathway, which is an indication for high photorespiratory flux in red algae. A plant-like photorespiratory metabolism with recruitment of peroxisomal GOX to improve the bottleneck reaction glycolate-to-glyoxylate conversion is an early evolutionary strategy to adapt to increasing O_2_ concentrations in the atmosphere. These findings are contrary to earlier assumptions that the plant-like photorespiration pathway appeared late and only among streptophytic green algae, which was discussed to be a crucial step for the later colonization of the continents by land plants (e.g. Becker, 2013).

## Supplementary data

Supplementary material is available at *JXB* online.

Table S1. Oligonucleotides used in this study.

Table S2. List of *A. thaliana* photorespiratory enzymes and identified homologous proteins in *C. merolae*.

Figure S1. Purification of recombinant CmGOX.

Figure S2. Chl *a* concentrations of WT and Δ*gox* mutants #43 and #46 during the CO_2_ shift experiment.

Figure S3. CO_2_-dependent growth of WT and Δ*gox* mutants #43 and #46.

Figure S4. Metabolite levels of WT and Δ*gox* mutants #43 and #46 during the CO_2_ shift experiment.

Supplementary Data

## References

[CIT0001] Atteia A Adrait A Brugiere S 2009 A proteomic survey of *Chlamydomonas reinhardtii* mitochondria sheds new light on the metabolic plasticity of the organelle and on the nature of the alpha-proteobacterial mitochondrial ancestor. Molecular Biology and Evolution 26, 1533–1548. 19349646 10.1093/molbev/msp068

[CIT0002] Bauwe H Hagemann M Fernie AR 2010 Photorespiration: players, partners and origin. Trends in Plant Science 15, 330–336. 20403720 10.1016/j.tplants.2010.03.006

[CIT0003] Becker B 2013 Snow ball earth and the split of Streptophyta and Chlorophyta. Trends in Plant Science 18, 180–183. 23102566 10.1016/j.tplants.2012.09.010

[CIT0004] Birmingham BC Coleman JR Colman B 1982 Measurement of photorespiration in algae. Plant Physiology 69, 259–262. 16662171 10.1104/pp.69.1.259 PMC426185

[CIT0005] Bradford M 1976 A rapid and sensitive method for the quantitation of microgram quantities of protein utilizing the principle of protein-dye binding. Analytical Biochemistry 72, 248–254. 942051 10.1016/0003-2697(76)90527-3

[CIT0006] Breuers FKH Bräutigam A Geimer S Welzel U Stefano G Renna L Brandizzi F Weber APM 2012 Dynamic remodeling of the plastid envelope membranes – a tool for chloroplast envelope in vivo localizations. Frontiers in Plant Science 3, 1–10. 22645566 10.3389/fpls.2012.00007 PMC3355811

[CIT0007] Colman B Balkos KD 2005 Mechanism of inorganic carbon acquisition in two *Euglena* species. Canadian Journal of Botany 83, 865–871.

[CIT0008] Czechowski T Stitt M Altmann T Udvardi MK 2005 Genome-wide identification and testing of superior reference genes for transcript normalization. Plant Physiology 139, 5–17. 16166256 10.1104/pp.105.063743 PMC1203353

[CIT0009] Eisenhut M Bauwe H Hagemann M 2007 Glycine accumulation is toxic for the cyanobacterium *Synechocystis sp*. strain PCC 6803, but can be compensated by supplementation with magnesium ions. FEMS Microbiology Letters 277, 232–237. 18031345 10.1111/j.1574-6968.2007.00960.x

[CIT0010] Eisenhut M Hocken N Weber APM 2015 Plastidial metabolite transporters integrate photorespiration with carbon, nitrogen, and sulfur metabolism. Cell Calcium 58, 98–104. 25465893 10.1016/j.ceca.2014.10.007

[CIT0011] Eisenhut M Kahlon S Hasse D Ewald R Lieman-Hurwitz J Ogawa T Ruth W Bauwe H Kaplan A Hagemann M 2006 The plant-like c2 glycolate cycle and the bacterial-like glycerate pathway cooperate in phosphoglycolate metabolism in cyanobacteria. Plant Physiology 142, 333–342. 16877700 10.1104/pp.106.082982 PMC1557606

[CIT0012] Eisenhut M Pick TR Bordych C Weber AP 2013 Towards closing the remaining gaps in photorespiration – the essential but unexplored role of transport proteins. Plant Biology 15, 676–685. 23199026 10.1111/j.1438-8677.2012.00690.x

[CIT0013] Eisenhut M Ruth W Haimovich M Bauwe H Kaplan A Hagemann M 2008 The photorespiratory glycolate metabolism is essential for cyanobacteria and might have been conveyed endosymbiontically to plants. PNAS 105, 17199–17204. 18957552 10.1073/pnas.0807043105 PMC2579401

[CIT0014] Engel N van den Daele K Kolukisaoglu U Morgenthal K Weckwerth W Parnik T Keerberg O Bauwe H 2007 Deletion of glycine decarboxylase in Arabidopsis is lethal under nonphotorespiratory conditions. Plant Physiology 144, 1328–1335. 17496108 10.1104/pp.107.099317 PMC1914133

[CIT0015] Fang W Si Y Douglass S Casero D Merchant SS Pellegrini M Ladunga I Liu P Spalding MH 2012 Transcriptome-wide changes in *Chlamydomonas reinhardtii* gene expression regulated by carbon dioxide and the CO_2_-concentrating mechanism regulator CIA5/CCM1. The Plant Cell 24, 1876–1893. 22634760 10.1105/tpc.112.097949 PMC3442575

[CIT0016] Fiehn O Kind T 2007 Metabolite profiling in blood plasma. Metabolomics - Methods in Molecular Biology 358, 3–17. 10.1007/978-1-59745-244-1_1 17035677

[CIT0017] Fujiwara T Kanesaki Y Hirooka S Era A Sumiya N Yoshikawa H Tanaka K Miyagishima S-Y 2015 A nitrogen source-dependent inducible and repressible gene expression system in the red alga *Cyanidioschyzon merolae* . Frontiers in Plant Science 6, 1–10. 26379685 10.3389/fpls.2015.00657 PMC4549557

[CIT0018] Gimmler H Kugel H Liebfritz D Mayer A 1988 Cytoplasmic pH of *Dunaliella parva* and *Dunaliella acidophila* as monitored by in vivo 31P-NMR spectroscopy and the DMO method. Physiologia Plantarum 74, 521–530.

[CIT0019] Giordano M Beardall J Raven JA 2005 CO_2_ concentrating mechanism in algae: mechanisms, environmental modulation, and evolution. Annual Review of Plant Biology 56, 99–131. 10.1146/annurev.arplant.56.032604.144052 15862091

[CIT0020] Grefen C Donald N Hashimoto K Kudla J Schumacher K Blatt MR 2010 A ubiquitin-10 promoter-based vector set for fluorescent protein tagging facilitates temporal stability and native protein distribution in transient and stable expression studies. Plant Journal 64, 355–365. 20735773 10.1111/j.1365-313X.2010.04322.x

[CIT0021] Hackenberg C Huege J Engelhardt A Wittink F Laue M Matthijs HC Kopka J Bauwe H Hagemann M 2012 Low carbon acclimation in carboxysome-less and photorespiratory mutants of the cyanobacterium *Synechocystis sp*. strain PCC 6803. Microbiology 158, 398–413. 22096149 10.1099/mic.0.054544-0

[CIT0022] Hackenberg C Kern R Hüge J Stal LJ Tsuji Y Kopka J Shiraiwa Y Bauwe H Hagemann M 2011 Cyanobacterial lactate oxidases serve as essential partners in N_2_ fixation and evolved into photorespiratory glycolate oxidases in plants. The Plant Cell 23, 2978–90. 21828292 10.1105/tpc.111.088070 PMC3180805

[CIT0023] Hagemann M Eisenhut M Hackenberg C Bauwe H 2010 Pathway and importance of photorespiratory 2-phosphoglycolate metabolism in cyanobacteria. Advances in Experimental Medicine and Biology 675, 91–108. 20532737 10.1007/978-1-4419-1528-3_6

[CIT0024] Hagemann M Fernie AR Espie GS Kern R Eisenhut M Reumann S Bauwe H Weber APM 2013 Evolution of the biochemistry of the photorespiratory C2 cycle. Plant Biology 15, 639–647. 23198988 10.1111/j.1438-8677.2012.00677.x

[CIT0025] Hagemann M Kern R Maurino VG Hanson DT Weber APM Sage RF Bauwe H 2016 Evolution of photorespiration from cyanobacteria to land plants considering protein phylogenies and acquisition of carbon concentrating mechanisms. Journal of Experimental Botany 67, 2963–2976. 26931168 10.1093/jxb/erw063

[CIT0026] Haimovich-Dayan M Lieman-Hurwitz J Orf I Hagemann M Kaplan A 2015 Does 2-phosphoglycolate serve as an internal signal molecule of inorganic carbon deprivation in the cyanobacterium *Synechocystis sp*. PCC 6803? Environmental Microbiology 17, 1794–1804. 25297829 10.1111/1462-2920.12638

[CIT0027] Heber U Krause GH 1980 Open question - what is the physiological role of photorespiration. Trends in Biochemical Sciences 5, 32–34.

[CIT0028] Husic DW Husic HD Tolbert NE Clanton CBJ 1987 The oxidative photosynthetic carbon cycle or C2 cycle. Critical Reviews in Plant Sciences 5, 45–100.

[CIT0029] Imamura S Terashita M Ohnuma M 2010 Nitrate assimilatory genes and their transcriptional regulation in a unicellular red alga *Cyanidioschyzon merolae*: Genetic evidence for nitrite reduction by a sulfite reductase-like enzyme. Plant and Cell Physiology 51, 707–717. 20375110 10.1093/pcp/pcq043

[CIT0030] Kehlenbeck P Coyal A Tolbert NE 1995 Factors affecting development of peroxisomes and glycolate metabolism among algae of different evolutionary lines of Prasinophyceae. Plant Physiology 109, 1363–1370. 12228674 10.1104/pp.109.4.1363 PMC157670

[CIT0031] Kern R Eisenhut M Bauwe H Weber APM Hagemann M 2013 Does the *Cyanophora paradoxa* genome revise our view on the evolution of photorespiratory enzymes? Plant Biology 15, 759–768. 23551942 10.1111/plb.12003

[CIT0032] Kozaki A Takeba G 1996 Photorespiration protects C3 plants from photooxidation. Nature 384, 557–560.

[CIT0033] Linka N Theodoulou FL Haslam RP Linka M Napier JA Neuhaus HE Weber AP 2008 Peroxisomal ATP import is essential for seedling development in *Arabidopsis thaliana* . Plant Cell 20, 3241–3257. 19073763 10.1105/tpc.108.062042 PMC2630453

[CIT0034] Matsuzaki M Misumi O Shin-I T 2004 Genome sequence of the ultrasmall unicellular red alga *Cyanidioschyzon merolae* 10D. Nature 428, 653–657. 15071595 10.1038/nature02398

[CIT0035] Meeks JC Castenholz RW 1971 Growth and photosynthesis in an extreme thermophile, *Synechococcus lividius* (Cyanophya). Archives of Microbiology 78, 25–41. 10.1007/BF00409086 4999393

[CIT0036] Mettler T Mühlhaus T Hemme D 2014 Systems analysis of the response of photosynthesis, metabolism, and growth to an increase in irradiance in the photosynthetic model organism *Chlamydomonas reinhardtii* . The Plant Cell 26, 2310–2350. 24894045 10.1105/tpc.114.124537 PMC4114937

[CIT0037] Minoda A Sakagami R Yagisawa F Kuroiwa T Tanaka K 2004 Improvement of culture conditions and evidence for nuclear transformation by homologous recombination in a red alga, *Cyanidioschyzon merolae* 10D. Plant and Cell Physiology 45, 667–671. 15215501 10.1093/pcp/pch087

[CIT0038] Moroney JV Jungnick N DiMario RJ Longstreth DJ 2013 Photorespiration and carbon concentrating mechanisms: two adaptations to high O_2_, low CO_2_ conditions. Photosynthesis Research 117, 121–131. 23771683 10.1007/s11120-013-9865-7

[CIT0039] Nakamura Y Kanakagiri S Van K He W Spalding MH 2005 Disruption of the glycolate dehydrogenase gene in the high-CO_2_-requiring mutant HCR89 of *Chlamydomonas reinhardtii* . Canadian Journal of Botany 83, 820–833.

[CIT0040] Norman EG Colman B 1991 Purification and characterization of phosphoglycolate phosphatase from the cyanobacterium *Coccochloris peniocystis* . Plant Physiology 95, 693–698. 16668041 10.1104/pp.95.3.693 PMC1077593

[CIT0041] Ogren WL Bowes G 1971 Ribulose diphosphate carboxylase regulates soybean photorespiration. Nature New Biology 230, 159–160. 5279476 10.1038/newbio230159a0

[CIT0042] Ohnuma M Yokoyama T Inouye T Sekine Y Tanaka K 2008 Polyethylene glycol (PEG)-mediated transient gene expression in a red alga, *Cyanidioschyzon merolae* 10D. Plant and Cell Physiology 49, 117–120. 18003671 10.1093/pcp/pcm157

[CIT0043] Pick TR Brautigam A Schulz MA Obata T Fernie AR Weber APM 2013 PLGG1, a plastidic glycolate glycerate transporter, is required for photorespiration and defines a unique class of metabolite transporters. Proceedings of the National Academy of Sciences U S A 110, 3185–3190. 10.1073/pnas.1215142110 PMC3581909 23382251

[CIT0044] Raven JA 2006 Sensing inorganic carbon: CO_2_ and HCO_3_ ^−^ . Biochemical Journal 395, e5–e7. 16703664 10.1042/BJ20060574 PMC1462711

[CIT0045] Renné P Dreßen U Hebbeker U Hille D Flügge UI Westhoff P Weber APM 2003 The Arabidopsis mutant dct is deficient in the plastidic glutamate/malate translocator DiT2. Plant Journal 35, 316–331. 12887583 10.1046/j.1365-313x.2003.01806.x

[CIT0046] Savir Y Noor E Milo R Tlusty T 2010 Cross-species analysis traces adaptation of Rubisco toward optimality in a low-dimensional landscape. Proceedings of the National Academy of Sciences U S A 107, 3475–3480. 10.1073/pnas.0911663107 PMC2840432 20142476

[CIT0047] Schwarte S Bauwe H 2007 Identification of the photorespiratory 2-phosphoglycolate phosphatase, PGLP1, in Arabidopsis. Plant Physiology 144, 1580–1586. 17478634 10.1104/pp.107.099192 PMC1914141

[CIT0048] Seckbach J 1995 The first eukaryotic cells - acid hot-spring algae. Journal of Biological Physics 20, 335–345.

[CIT0049] Simon P 2003 Q-Gene: processing quantitative real-time RT-PCR data. Bioinformatics 19, 1439–1440. 12874059 10.1093/bioinformatics/btg157

[CIT0050] Somerville CR 2001 An early Arabidopsis demonstration. Resolving a few issues concerning photorespiration. Plant Physiology 125, 20–24. 11154287 10.1104/pp.125.1.20 PMC1539316

[CIT0051] Stabenau H 1974 Verteilung von Microbody-Enzymen aus Chlamydomonas in Dichtegradienten [Distribution of microbody enzymes in density gradients]. Planta 118, 35–42. 24442197 10.1007/BF00390501

[CIT0052] Stabenau H Winkler U Säftel W 1993 Localization of glycolate dehydrogenase in two species of *Dunaliella* . Planta 191, 362–364.

[CIT0053] Suzuki K Mamedov TG Ikawa T 1999 A mutant of *Chlamydomonas reinhardtii* with reduced rate of photorespiration. Plant and Cell Physiology 40, 792–799.

[CIT0054] Takahashi S Bauwe H Badger M 2007 Impairment of the photorespiratory pathway accelerates photoinhibition of photosystem II by suppression of repair but not acceleration of damage processes in Arabidopsis. Plant Physiology 144, 487–494. 17400706 10.1104/pp.107.097253 PMC1913796

[CIT0055] Timm S Florian A Jahnke K Nunes-Nesi A Fernie AR Bauwe H 2011 The hydroxypyruvate-reducing system in Arabidopsis: multiple enzymes for the same end. Plant Physiology 155, 694–705. 21205613 10.1104/pp.110.166538 PMC3032460

[CIT0056] Uemura K Anwaruzzaman Miyachi S Yokota A 1997 Ribulose-1,5-bisphosphate carboxylase/oxygenase from thermophilic red algae with a strong specificity for CO_2_ fixation. Biochemical and Biophysical Research Communications 233, 568–571. 9144578 10.1006/bbrc.1997.6497

[CIT0057] Voll LM Jamai A Renné P Voll H McClung CR Weber APM 2006 The photorespiratory Arabidopsis *shm1* mutant is deficient in SHM1. Plant Physiology 140, 59–66. 16339799 10.1104/pp.105.071399 PMC1326031

[CIT0058] Watanabe S Ohnuma M Sato J Yoshikawa H Tanaka K 2011 Utility of a GFP reporter system in the red alga *Cyanidioschyzon merolae* . The Journal of General and Applied Microbiology 57, 69–72. 21478650 10.2323/jgam.57.69

[CIT0059] Weber A Flügge UI 2002 Interaction of cytosolic and plastidic nitrogen metabolism in plants. Journal of Experimental Botany 53, 865–874. 11912229 10.1093/jexbot/53.370.865

[CIT0060] Zelitch I Schultes NP Peterson RB Brown P Brutnell TP 2009 High glycolate oxidase activity is required for survival of maize in normal air. Plant Physiology 149, 195–204. 18805949 10.1104/pp.108.128439 PMC2613714

[CIT0061] Zenvirth D Volokita M Kaplan A 1985 Photosynthesis and inorganic carbon accumulation in the acidophilic alga *Cyanidioschyzon merolae* . Plant Physiology 77, 237–239. 16664017 10.1104/pp.77.1.237 PMC1064490

