# Supplementary material for: Photorespiratory glycolate oxidase is essential for the survival of the red alga Cyanidioschyzon merolae under ambient CO2 conditions
Source: J Exp Bot. 2016 Mar 19;67(10):3165–75. doi: 10.1093/jxb/erw118 (PMC4867895; doi:10.1093/jxb/erw118)
Supplement: Supplementary Data [file supp_67_10_3165__index.html]

Photorespiratory glycolate oxidase is essential for the survival of the red alga Cyanidioschyzon merolae under ambient CO2 conditions — Photorespiratory glycolate oxidase is essential for the survival of the red alga Cyanidioschyzon merolae under ambient CO2 conditions — Supplementary Data 

# Photorespiratory glycolate oxidase is essential for the survival of the red alga *Cyanidioschyzon merolae* under ambient CO2 conditions

## Supplementary Data

Data files

- supplementary\_tables\_S1\_S2\_figures\_S1\_S4.pdf - Supplementary Data
