# Supplementary material for: Photorespiratory glycolate oxidase is essential for the survival of the red alga Cyanidioschyzon merolae under ambient CO2 conditions
Source: J Exp Bot. 2016 Mar 19;67(10):3165–75. doi: 10.1093/jxb/erw118 (PMC4867895; doi:10.1093/jxb/erw118)
Supplement: Supplementary Data [file supp_erw118_supplementary_tables_S1_S2_figures_S1_S4.pdf]

SUPPLEMENTARY MATERIAL

Photorespiratory glycolate oxidase is essential for survival of the red alga *Cyanidioschyzon merolae* under ambient CO_2 conditions

Nadine Rademacher, Ramona Kern, Takayuki Fujiwara, Tabea Mettler-Altmann, Shin-ya Miyagishima, Martin Hagemann, Marion Eisenhut, Andreas PM Weber

Table S1: Oligonucleotides used in this study. Restriction sites are given in bold. Underlines indicate adaptor sequences for In-Fusion reaction.

**Oligonucleotide**	**Sequence (5´ -> 3`)**
P1	ccacaattgATGGTTGAGAAGCCAGC
P2	ccaccatggGCTCAGAGTTTGCTCTGC
P3	ccagtttaaacTTACAATACCGATAGATGAGTTTCGA
P4	ccaactagtGGTCAACGAACGAAGAAACACA
qRT-CmGOX-fw	GCCTAGCAGTTGATGGCGA
qRT-CmGOX-rev	CCCGAACGACGAGATCTCTC
qRT-CmBlack-fw	TCACGCAAAACAACATCCAT
qRT-CmBlack-rev	TCAACAGCGTTGAATCGAAG
qRT-CmrbcL-fw	GTGCCACAGCTAACCGTGTA
qRT-CmrbcL-rev	AGCGGTTTGAAGAGGACCAC
CMQ436C-EcoRI-fw	**GAATTC**ATGGTTGAGAAGCCAGCAG
CMQ436C-SalI-rv	**GTCGAC**TTAGAGTTTGCTCTGCATC
GOX_KO_F1	ACCATCACCATCACGTCGAGAACTGGAACTTGTCC
GOX_KO_R1	AAGCTCAGCTAATTACGATGTTGCAGTGCGTACG
GOX_KO_F2	AGTCAGCTGCTAGGGTGAGCGCAGGCTGGAG
GOX_KO_R2	TTCGCCCCTCAGTTCCTCTACTTTGTTTCGAATACTGGTAAAACAA
URA_F	GAACTGAGGGGCGAACGCA
URA_R	CCCTAGCAGCTGACTGTATC
GOX_KO_F3	GTCGAGAACTGGAACTTGTCC
GOX_KO_R3	CGATGTTGCAGTGCGTACG
GOX_KO_F4	CGCAGGCATTCAGGGCAG
GOX_KO_R4	CGCGTGATGGATCGCATTGC

GOX_KO_F5	CGTTTCGACGTCTACGCTTCC
GOX_KO_R5	CTCGAGGCAGTCGATGGTG
CMQ432C-F	TCTACGCTGCCGGAGATTG
CMQ432C-R	GCCATACCAAAGCCAGAACG

Table S2: List of *A. thaliana* photorespiratory enzymes and identified homologous proteins in *C. merolae*.

**Photorespiratory enzyme**	***A. thaliana***	***C. merolae***	**Identity (%)**	**Similarity (%)**
2-PG phosphatase*	AT5G36700	CMR421C	56	68
Glycolate oxidase	AT3G14420	CMQ436C	59	78
	AT4G18360		59	76
	AT3G14415		59	77
Catalase 2	AT4G35090	CMI050C	61	76
Serine:glyoxylate aminotransferase*	AT2G13360	CMS429C	51	68
Glutamate:glyoxylate aminotransferase*	AT1G23310	CMM066C	38	54
T-protein*	AT1G11860	CMG086C	43	59
	AT1G60990		21	39
P-protein	AT4G33010	CMR282C	56	69
	AT2G26080		56	70
H-protein	AT2G35370	CMF098C	50	67
	AT2G35120		52	68
	AT1G32470		51	66
L-protein	AT3G17240	CMM299C	58	74
	AT1G48030		58	74
Serine hydroxymethyl transferase*	AT4G37930	CMO142C	62	78
Hydroxypyruvate reductase 1*	AT1G68010	CMS425C	56	74
Hydroxypyruvate reductase 2	AT1G79870	CMQ289C	39	55
	AT1G68010		38	54
Glycerate kinase*	AT1G80380	CMK141C	37	53

*best bidirectional hit

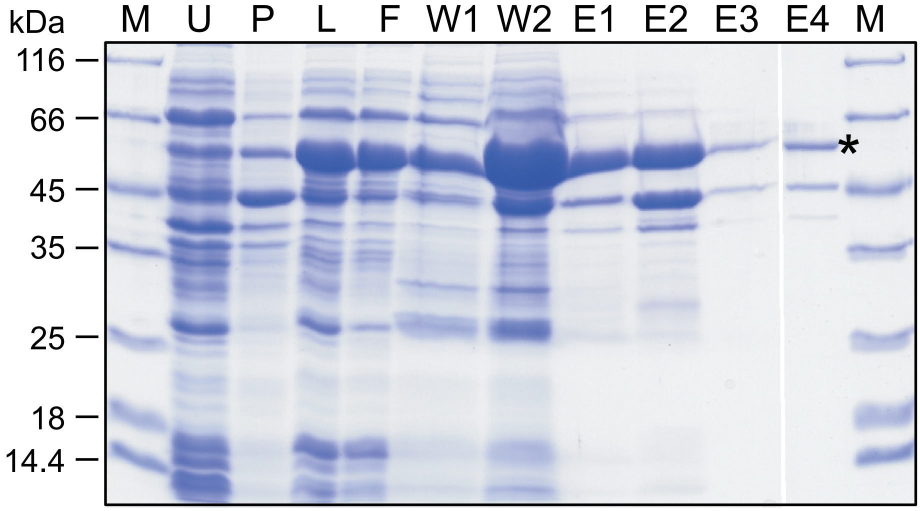

Figure S1: Purification of recombinant CmGOX. N-terminally His-tagged CmGOX (*) was purified via Ni-NTA- affinity chromatography. The desalted elution fraction (E4) was used for *in vitro* enzyme assays of putative GOX/LOX activity. M, Protein Molecular weight marker; U, uninduced crude cell extract; P, Cell-debris obtained after cell disruption and centrifugation; L, Lysat; F, Flow-through of lysat; W1 and W2, Washing flow-through; E1-E3, Elution fractions, which were combined and desalted using PD-10 columns giving E4.

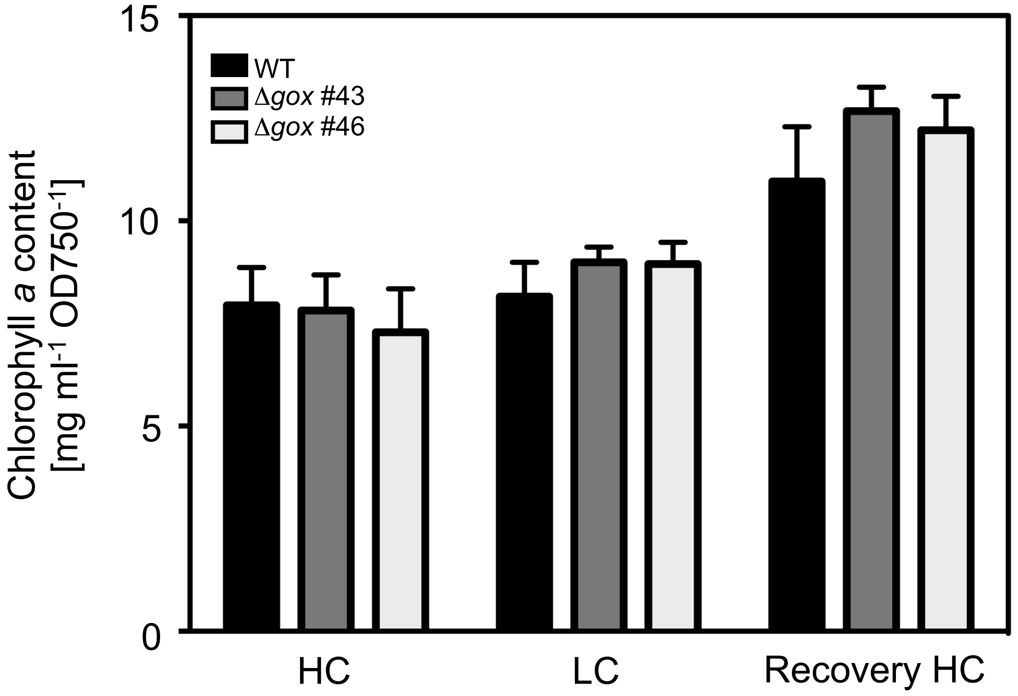

Figure S2: Chlorophyll *a* concentrations of WT and Δ*gox* mutants #43 and #46 during the CO_2 shift experiment. No significant differences ($P < 0.05$) were determined with the two-tailed Student´s t-test.

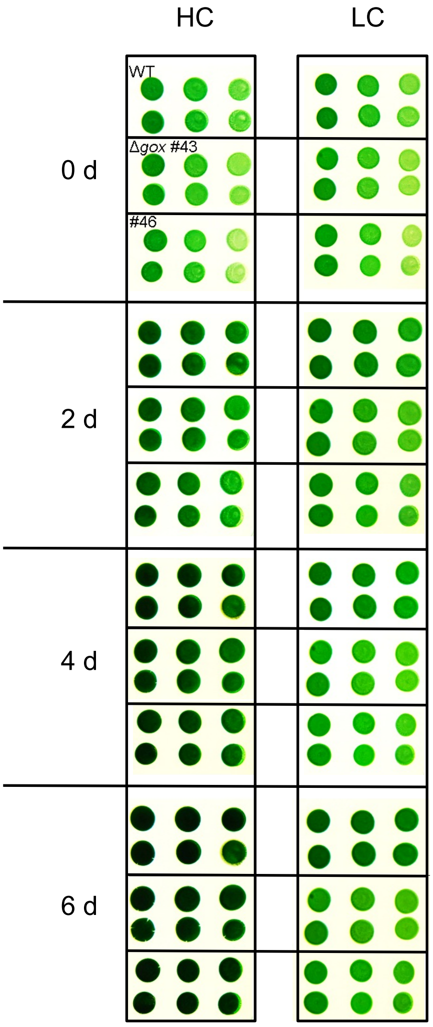

Figure S3: CO_2-dependent growth of WT and Δ*gox* mutants #43 and #46. All cell lines were pre-cultured in 2xMA medium for 1 d under HC conditions. Then cultures were adjusted to a cell density OD_{750} of 2.5, 0.83 and 0.28 and spotted onto a starch bed on solidified 2xMA plates. The plates were incubated for 4 d under HC conditions and continuous light (20 µmol photons m^{-2} s^{-1}) and then shifted to LC conditions. Growth was photographically documented after 0, 2, 4 and 6 d.

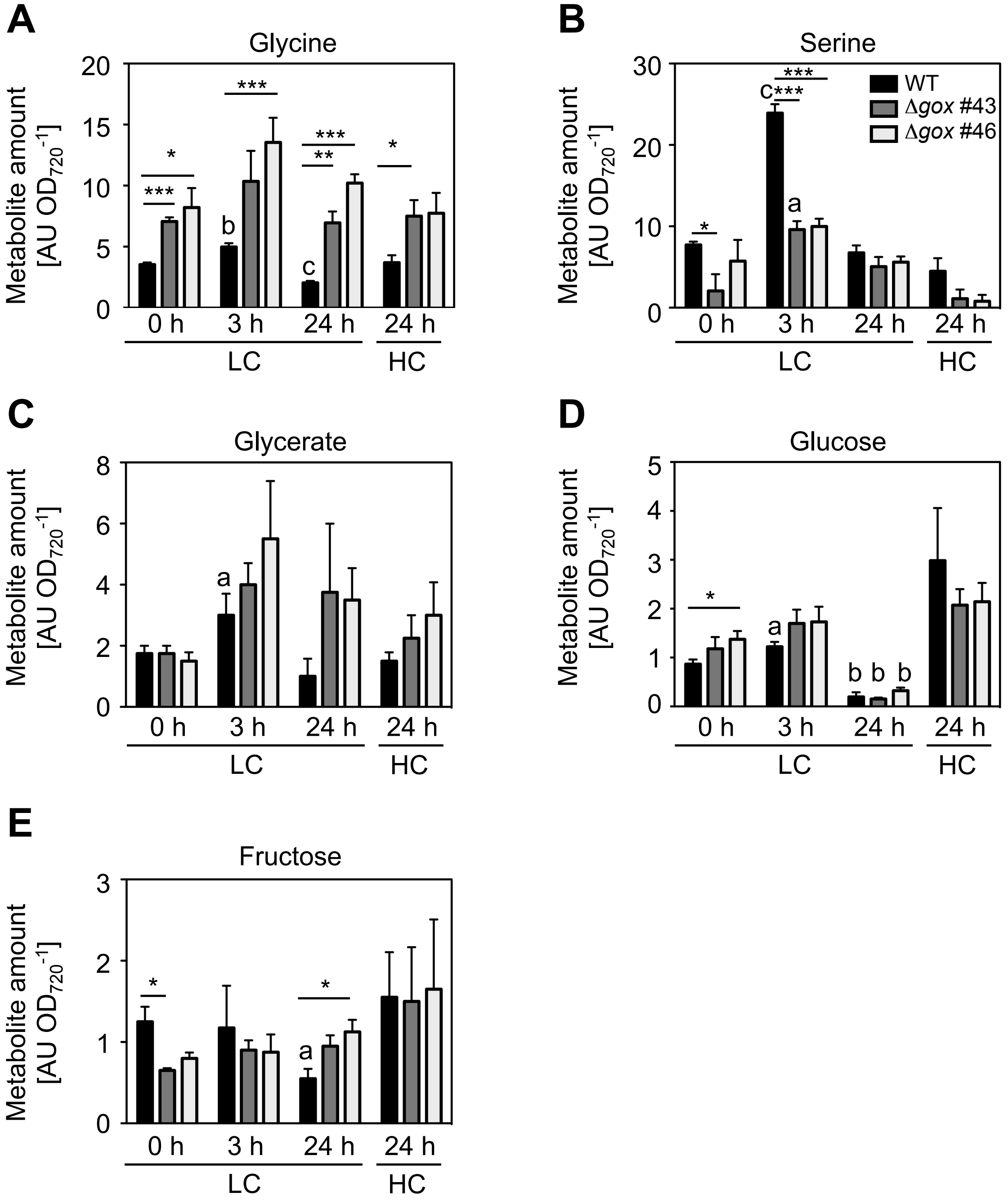

Figure S4: Metabolite levels of WT and Δ*gox* mutants #43 and #46 during the CO_2 shift experiment. Glycine (A), serine (B), glycerate (C), glucose (D), and fructose (E) levels of WT and Δ*gox* mutants #43 and #46 were determined by GC-MS and HPLC, respectively, before (0 h), 3 h and 24 h after the shift from HC (5% CO_2) to LC (0.04% CO_2) conditions, and after a 24 h recovery phase under HC conditions. Shown are mean values and standard errors of four biological replicates. Significant differences between lines were determined with the two-tailed Student´s t-test and are indicated as: $P < 0.05$ (*), $P < 0.01$ (**), $P < 0.001$ (***). Significant differences to initial levels (LC, 0 h) are indicated as: a ($P < 0.05$), b ($P < 0.01$) and c ($P < 0.001$).
